# Hydrochlorate of Cocaine as a Local Anæsthetic

**Published:** 1885-03-20

**Authors:** W. L. Bullard

**Affiliations:** Columbus, Ga.


### HYDROCHLORATE OF COCAINE AS A LOCAL ANÆSTHETIC.

By W. L. BULLARD, COLUMBUS, GA.

Being in Europe at the time this new anæsthetic was introduced, I became interested in noticing the effect as applied to mucous membranes. At the clinics of Mr. Lawson Critchet, Tweedy and Nettleship, held at the Royal London Ophthalmic Hospital, I witnessed under the direction of these surgeons, various operations on the eye, with the solution of cocaine of different strengths.

In the minor operations, such as extraction, iridectomies, Salmisch's operation, convergent strabismus, tattooing cornea, extracting foreign bodies, etc., the two-per-cent. solution of Dr. Koller was at first used. increased afterwards to a four-per-cent. solution. In major operations, such as excisions, divergent strabismus, mucocele, cauthoplasty, chalazion, etc., a ten and twenty-per-cent. solution was used.

After noting the effect in different operations of the solutions of various strengths, the universal conclusions arrived at were:

1. A 4% solution produced complete anæsthesia in all operations involving conjunctiva and cornea.

2. That a 4% solution offered all the advantages, in operations involving the deeper tissues that can be obtained from the instillation of a stronger solution.

3. That in operations involving iris and division of tendons (tenotomy, etc.) quite as many patients claim that they feel more or less pain as there are those who do not.

4 That hydro. chlo. cocaine is not a perfect anæsthetic for operations involving the deeper tissues of the eye.

In connection with this new agent, it is proper to mention that I have found a fifteen to twenty-per-cent. solution effective in operations of the throat and nose.

I select from my own practice the following cases operated on since my return home:

Miss L. H., æt. 12, ulcer of cornea with onyx. Performed Salmisch's operation; puncture and counter puncture were painless; extracting pus gave "slight pain," though she betrayed no evidence of it.

Mrs. C. J. S., æt. 35, Georgia, opacity of cornea, both eyes. Performed iridectomy; section through conjunctiva and cornea "perfectly painless," but on removal of iris patient complained of some pain.

Mrs. M. A. G., æt. 33, Alabama, acute glaucoma. Performed iridectomy; the sclerotic incision painless, clipping of iris compared to a "pin scratch."

J. M. C., æt. 65, senile cataract. The whole operation painless, save a slight pain in clipping iris.

Miss L. H. F., divergent strabismus. Division of tendon, with advancement of muscle, etc. Patient gave evidence of pain when tendon was divided; also complained of "great discomfort" in the manipulation necessary to advancement of the muscle.

In cases reported (except one) I used the gelatine disc of hydro. chlo. cocaine, equal to 4% solution; discs prepared by Savury & Moore, London. It is claimed for these discs that they retain their strength indefinitely.

Columbus, Ga., February 1, 1885.

